# Title of “Ambassador of Clinical Psychology and Psychological Treatment” Awarded to Peter Fonagy

**DOI:** 10.32872/cpe.7781

**Published:** 2022-03-31

**Authors:** Martin Debbané

**Affiliations:** 1 Faculty of Psychology and Educational Sciences, University of Geneva, Geneva, Switzerland; 2 Department of Clinical, Educational, and Health Psychology, University College London, London, United Kingdom

**Professor Peter Fonagy (OBE**) leads a career in clinical psychology that epitomizes an integrative approach to the psychological care for children, adolescents and adults, with a continued determination to alleviate mental pain in those suffering from often chronic psychological distress. Driven by the ambition of increasing access to quality care for the vulnerable, he has occupied a number of key national leadership positions in the UK, including Chair of the Outcomes Measurement Reference Group at the Department of Health, Chair of two NICE Guideline Development Groups, Chair of the Strategy Group for National Occupational Standards for Psychological Therapies and co-chaired the Department of Health's Expert Reference Group on Vulnerable Children.

**Figure:**
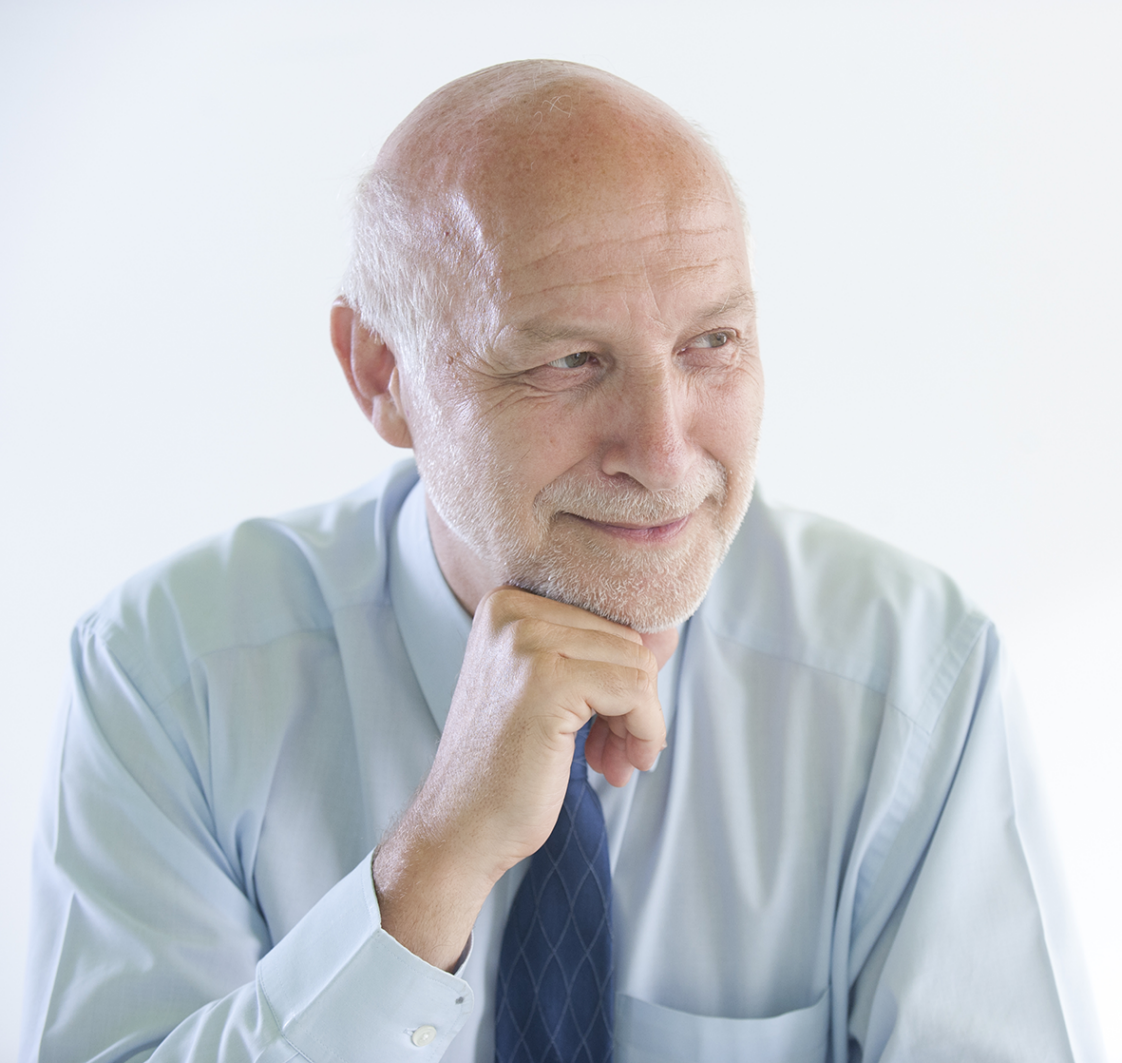
Peter Fonagy (2013)

His clinical interests centre on issues of early attachment relationships, resilience, social cognition, borderline personality disorder and violence. Drawing from psychoanalysis, developmental psychology, attachment theory as well as cognitive and affective neuroscience, Peter Fonagy puts forward a clinical approach based on evidence as well as best practice, closely articulated to the most recent developments in research on psychopathology and psychotherapy. A major focus of his contribution has been an innovative research-based psychodynamic therapeutic approach, mentalization-based treatment, which was developed in collaboration with a number of clinical sites in the UK and USA. He has published over 500 scientific papers, 260 chapters and has authored or co-authored 19 books. Embracing communication and collaboration over competition and hostility between different theoretical frameworks in psychotherapy, his most popular books include “What Works for Whom” and “Affect regulation, mentalization and the development of the self”, which collectively have attracted over ten thousand citations.

His recognition as a scientist include Fellow of the British Academy, the Academy of Medical Sciences, the Academy of Social Sciences and the American Association for Psychological Science, and he was elected to Honorary Fellowship by the American College of Psychiatrists. He has received Lifetime Achievement Awards from several national and international professional associations including the British Psychological Society, the International Society for the Study of Personality Disorder, the British and Irish Group for the Study of Personality Disorder, the World Association for Infant Mental Health and was in 2015 the first UK recipient of the Wiley Prize of the British Academy for Outstanding Achievements in Psychology by an international scholar.

Peter Fonagy’s academic achievements are recognized not only in the UK and in Europe, but also at the international level. He is currently Head of the Division of Psychology and Language Sciences at University College London; Chief Executive of the Anna Freud National Centre for Children and Families, London; Consultant to the Child and Family Programme at the Menninger Department of Psychiatry and Behavioural Sciences at Baylor College of Medicine; and holds visiting professorships at Yale and Harvard Medical Schools.

Most importantly perhaps, Peter Fonagy’s work influences hundreds of clinical psychologists across many different theoretical approaches to reflect on the common factors leading to salutogenesis, that is, the psychological mechanisms which sustain mental health in the face of the regular and more impactful challenges individuals face across the lifespan. Beyond individual and group psychotherapy, Peter Fonagy advocates for a social and political approach to mental health, and his work underlines the responsibilities we carry as families, communities and political entities to strive to care for each other and be kind to one another. Profound humanism can be experienced from Peter Fonagy’s approach to clinical psychology. He has agreed to share and defend these values as a dedicated ambassador to the European Association for Clinical Psychology and Allied Disciplines.

 

**Details on his life and professional trajectories in the media**
- https://www.theguardian.com/society/2019/apr/27/peter-fonagy-refugee-child-psychologist-anna-freud-centre
- https://www.bbc.co.uk/sounds/play/m000dpj2

***Citation from an interview with E. L. Jurist (2010, p. 7)***

P. F. *(…) when we understand the mechanism of a disorder at the level of biology, at the level of neuroscience, we will also understand (…) that the only way to alter those things will be psychological. They will be much more targeted, better targeted, but they will be psychological interventions.*

E. L. J. *So there’s something ineradicable about the role of psychology.*

P. F. *We are here for the duration.*
